# Dual inhibition of DNMTs and EZH2 can overcome both intrinsic and acquired resistance of myeloma cells to IMiDs in a cereblon‐independent manner

**DOI:** 10.1002/1878-0261.12157

**Published:** 2017-12-30

**Authors:** Konstantinos Dimopoulos, Alexandra Søgaard Helbo, Helga Fibiger Munch‐Petersen, Lene Sjö, Jesper Christensen, Lasse Sommer Kristensen, Fazila Asmar, Niels Emil Ulrich Hermansen, Casey O'Connel, Peter Gimsing, Gangning Liang, Kirsten Grønbæk

**Affiliations:** ^1^ Department of Hematology Rigshospitalet, University Hospital Copenhagen Denmark; ^2^ Biotech Research and Innovation Centre (BRIC) University of Copenhagen Denmark; ^3^ Department of Pathology Rigshospitalet, University Hospital Copenhagen Denmark; ^4^ Interdisciplinary Nanoscience Center (iNANO) Aarhus University Denmark; ^5^ Department of Urology and Hematology USC Norris Comprehensive Cancer Center University of Southern California Los Angeles CA USA

**Keywords:** 5‐azacytidine, cereblon, epigenetics, immunomodulatory drugs, multiple myeloma

## Abstract

Thalidomide and its derivatives, lenalidomide and pomalidomide (also known as IMiDs), have significantly changed the treatment landscape of multiple myeloma, and the recent discovery of cereblon (CRBN) as their direct biological target has led to a deeper understanding of their complex mechanism of action. In an effort to comprehend the precise mechanisms behind the development of IMiD resistance and examine whether it is potentially reversible, we established lenalidomide‐resistant (‐LR) and pomalidomide‐resistant (‐PR) human myeloma cell lines from two IMiD‐sensitive cell lines, OPM2 and NCI‐H929, by continuous culture in the presence of lenalidomide or pomalidomide for 4–6 months, until acquirement of stable resistance. By assessing genome‐wide DNA methylation and chromatin accessibility in these cell lines, we found that acquired IMiD resistance is associated with an increase in genome‐wide DNA methylation and an even greater reduction in chromatin accessibility. Transcriptome analysis confirmed that resistant cell lines are mainly characterized by a reduction in gene expression, identifying SMAD3 as a commonly downregulated gene in IMiD‐resistant cell lines. Moreover, we show that these changes are potentially reversible, as combination of 5‐azacytidine and EPZ‐6438 not only restored the observed accessibility changes and the expression of SMAD3, but also resensitized the resistant cells to both lenalidomide and pomalidomide. Interestingly, the resensitization process was independent of CRBN. Our data suggest that simultaneous inhibition of DNA methyl transferases and EZH2 leads to an extensive epigenetic reprogramming which allows myeloma cells to (re)gain sensitivity to IMiDs.

**Abbreviations**
CRBN: cereblon
DNMT1: DNA methyl transferase 1
dscDNA: double‐stranded cDNA
MM: multiple myeloma
MS‐MCA: methylation‐specific melting curve analysis
NoE: no enzyme
SAM: S‐adenosylmethionine

## 1 Introduction

The introduction of novel agents for the treatment of multiple myeloma (MM), mainly proteasome inhibitors and immunomodulatory agents (IMiDs), has significantly improved the survival rates of the patients, and both classes of drugs stand as the main treatment options for MM (Mahindra *et al*., 2012). The class of IMiDs comprises thalidomide, and its derivatives, lenalidomide and pomalidomide, and even though their antimyeloma activity had been confirmed in several clinical trials, it was not until recently that their precise mechanism of action was deciphered.

A groundbreaking study identified cereblon (CRBN) as the direct target of thalidomide, suggesting a model that explained the drug's teratogenic effects (Ito *et al*., 2010). Further studies have since confirmed that not only is CRBN the direct target of thalidomide, but also that of lenalidomide and pomalidomide, and that its expression is required for the antimyeloma effect of these drugs (Lopez‐Girona *et al*., 2012; Schuster *et al*., 2014; Zhu *et al*., 2011). CRBN has been shown to be part of an E3 ubiquitin ligase complex (also known as CRL4^CRBN^) together with CUL4A, DDB1, and RBX1, where it serves as a substrate receptor (Angers *et al*., 2006; Fischer *et al*., 2014). The CRBN‐mediated mechanism of action of IMiDs is quite distinctive, as the interaction of IMiDs with CRBN results in increased affinity of CRBN toward novel substrates, such as the transcription factors Ikaros (IKZF1) and Aiolos (IKZF3), which subsequently get ubiquitinated by CRL4^CRBN^ and degraded in the proteasome, leading among others to reduced expression of IRF4 in plasma cells and increased expression of IL‐2 in T cells (Fischer *et al*., 2014; Gandhi *et al*., 2013a; Krönke *et al*., 2014; Lu *et al*., 2013). Degradation of IKZF1 and IKZF3 by CRBN seems, however, to be myeloma specific, as other CRBN neosubstrates have been associated with different diseases, such as casein kinase 1 alpha (CK1α) in myelodysplastic syndromes with the deletion of chromosome 5q (Krönke *et al*., 2015).

Interestingly, low expression of CRBN, both at mRNA and at protein level, has consistently been found to be associated with a worse response to IMiDs or even resistant/refractory disease, suggesting a role as a potential predictive biomarker for response to treatment to IMiDs (Broyl *et al*., 2013; Heintel *et al*., 2013; Huang *et al*., 2014; Ren *et al*., 2015; Schuster *et al*., 2014; Zhu *et al*., 2011). However, the mechanisms that regulate CRBN expression still remain elusive. It has been shown that mutations of CRBN are infrequent in MM, thus suggesting a potential epigenetic component (Egan *et al*., 2013; Thakurta *et al*., 2014).

In this study, we investigated the regulation of CRBN expression in human myeloma cell lines with both intrinsic and acquired resistance to IMiDs. We examined whether epigenetic mechanisms such as DNA methylation, repressive histone marks (such as histone lysine 27 trimethylation – H3K27me3), or nucleosome occupancy can potentially regulate the expression of CRBN in these cell lines. By performing Acce*SssI*ble together with RNA‐seq, we assessed the changes in DNA methylation and chromatin accessibility, as well as gene expression, which were associated with acquired IMiD resistance. Finally, we tested whether resensitization of resistant MM cell lines to IMiDs is feasible through epigenetic reprogramming by epigenetic modulators either as single agents or in combination.

## 2 Methods

### 2.1 Cell culture and treatments

The human myeloma cell lines JJN3, OPM2, RPMI‐8226, U266, KMS12‐BM, and NCI‐H929 were purchased from DSMZ (Leibniz Institute DSMZ–German Collection of Microorganisms and Cell Cultures, Braunschweig, Germany) and were grown using the recommended cell culture medium by DSMZ, at 37 °C with 5% CO_2_ in a humidified atmosphere and under sterile conditions. Cell density and viability were determined using the cell counter NC‐250 (Chemometec, Lillerød, Denmark). For the development of IMiD‐resistant cell lines, we treated OPM2 and NCI‐H929 continuously with increasing doses of either lenalidomide (Selleck Chemicals, Houston, TX, USA) or pomalidomide (Selleck Chemicals) for 4–6 months, until cell viability and proliferation were not affected, as previously described (Lopez‐Girona *et al*., 2012). The starting dose of the compounds was 1 μm for OPM2 and 0.1 μm for NCI‐H929. Fresh lenalidomide or pomalidomide was added every 2–3 days in the cell culture and the dose was escalated in several steps (to a final dose of 10 μm) whenever the cells consecutively exhibited viability above 90% (as calculated by the NC250) in the presence of the drug. The cells were not treated with any compounds for a minimum of 7 days before all the experiments performed in this study.

Other chemotherapeutic agents used in the cell culture in this study were 5‐azacytidine (MedChem Express, Monmouth Junction, NJ, USA) and EPZ‐6438 (MedChem Express).

### 2.2 Cell proliferation and apoptosis assays

Cell proliferation was determined using the XTT Cell Proliferation Kit II (Roche, Mannheim, Germany). Briefly, cells were seeded in different concentrations (between 2 × 10^5^ and 4 × 10^5^ cells·mL^−1^) in 96‐well plates and were left untreated (control) or treated with serial doses of lenalidomide and pomalidomide. Following incubation for 3 days, 100 μL of XTT Labeling Reagent was added in each well, and after a further incubation of 2–4 h, absorbance was read at 450 and 650 nm (reference wavelength). Generation of dose–response curves and calculation of IC_50_ values for lenalidomide and pomalidomide for each cell line were performed using graphpad prism 6 software (GraphPad Software, Inc., La Jolla, CA, USA).

For cell apoptosis, we used the FITC Annexin V Apoptosis Detection Kit I (BD Biosciences, San Jose, CA, USA), according to the manufacturers' protocol. All samples were analyzed in a FACS Calibur (BD Biosciences). All experiments were performed in a triplicate and repeated at least once.

### 2.3 Patient samples

A total of 89 bone marrow samples were obtained from patients with MM diagnosed with MM between 2007 and 2009, according to the IMWG criteria, at the Department of Hematology, Rigshospitalet, Denmark. From these samples, 48 were obtained at diagnosis and 41 at relapse. For all the samples, the CD138+ plasma cells were isolated from the mononuclear cell population from each bone marrow sample with the use of a RoboSep (Stem Cell Technologies, Vancouver, BC, Canada). All included patients provided written consent in accordance with the Helsinki Declaration. The Danish National Ethical Committee approved the conduction of this study.

### 2.4 Nucleic acid extraction

Total DNA and RNA were isolated from each cell line using All Prep DNA/RNA/miRNA Universal kit (Qiagen, Hilden, Germany) according to the manufacturers' protocol. The quantity (260 nm) and quality (260/280 and 260/230 ratios) of total DNA and RNA were measured by spectrophotometry on a NanoDrop‐1000 spectrophotometer (Thermo Scientific, Waltham, MA, USA).

### 2.5 Methylation‐specific melting curve analysis (MS‐MCA)

Methylation screening for the promoter of *CRBN* was performed by MS‐MCA as described, using 1 μg of bisulfite‐converted DNA as a template (Guldberg *et al*., 2002). The primer sequences for CRBN used in this assay were as follows: CRBN‐F: 5′‐TGTTTATTAGTAAAGGAGGTTGGGATAGG‐3′, CRBN‐R: 5′‐CCCTCCTAAACCATCACTTTCAAACTT‐3′.

The melting peaks were calculated using the lightcycler 480 Software Release 1.5.0SP3. SssI‐treated DNA (Millipore, Burlington, MA, USA) and unmethylated genomic DNA (Roche) were also bisulfite‐converted and used as positive (methylated) and negative (unmethylated) control, respectively.

### 2.6 Quantitative PCR

One microgram of RNA was reverse‐transcribed using SuperScript III First Strand Synthesis kit (Life Technologies, Carlsbad, CA, USA) according to the manufacturers' instructions. Subsequently, the samples were diluted 1 : 10 in DNase/RNase‐free water and qPCR was performed in duplicate in a 96‐well format using 5 μL of cDNA and SYBR Green I Master Mix (Roche) to a total volume of 20 μL. The PCR amplification was carried out with the following PCR cycling conditions: one cycle of 95 °C for 10 min, followed by 45 cycles of 95 °C for 10 s, 60 °C for 20 s, and 72 °C for 30 s, and one cycle of 72 °C for 10 min, on a LightCycler® 480 instrument II (Roche Diagnostics). All the qPCR primers used in this study are shown in Table 1. Stably expressed reference genes were determined by importing *C*_t_ values transformed into a linear‐scale expression quantities, and raw *C*_t_ values into the NormFinder (Andersen *et al*., 2004) and GeNorm algorithms (Vandesompele *et al*., 2002), respectively. A total of 10 potential reference genes (*GAPDH, ACTB, SF3A1, PUM1, IPO8, UBC, ESP, B2M, TBP,* and *HPRT1*), many of which have been shown to be stably expressed in cancer (Søes *et al*., 2013), were tested. *SF3A1* and *PUM1* were determined as the best reference genes by both algorithms (data not shown) and were therefore used for normalization of all qPCR data in this study. Relative gene expression was calculated by using the comparative threshold method ($2^{-\Delta \Delta C_{t}}$; Livak and Schmittgen, 2001).

**Table 1:** All the primer sequences used in qPCR experiments in this study

Gene	Forward primer	Reverse primer
*GAPDH*	5′‐CCACTCCTCCACCTTTGACG‐3′	5′‐TGTCATACCAGGAAATGAGCTTG‐3′
*ACTB*	5′‐CTGGACGGTGAAGGTGACA‐3′	5′‐AAGGGACTTCCTGTAACAATG‐3′
*SF3A1*	5′‐TCCATCCGTGAGAAGCAGAGC‐3′	5′‐TCTGGATCTCCTCCTCACCG‐3′
*HPRT1*	5′‐TGACACTGGCAAAACAATGCA‐3′	5′‐GGTCCTTTTCACCAGCAAGCT‐3′
*UBC*	5′‐TTAGGACGGGACTTGGGTGA‐3′	5′‐CCTGTTCCGCTCTCTGGAAA‐3′
*ESD*	5′‐GCCTTTAGTGGATATTTGGGAACA‐3′	5′‐GGGTAGCATCATAAGCCTTCCA‐3′
*B2M*	5′‐TGCTGTCTCCATGTTTGATGTATCT‐3′	5′‐TCTCTGCTCCCCACCTCTAAGT‐3′
*PUM1*	5′‐CATGCCAGGTTATCCGGTGT‐3′	5′‐GCGCCTGCATTCACTACAAG‐3′
*TBP*	5′‐TGCACAGGAGCCAAGAGTGAA‐3′	5′‐CACATCACAGCTCCCCACCA‐3′
*IPO8*	5′‐CCTCACAACCCTGGACCTATC‐3′	5′‐ACATCTTCCGGTCATGATGC‐3′
*CRBN*	5′‐CCAGTCTGCCGACATCACAT‐3′	5′‐GTCATCGTGCAAAGTCCTGC‐3′
*IKZF1*	5′‐ACAGCAAAGCTCCAAGAGTGA‐3′	5′‐TCCCCATTCATTTCACAGGCA‐3′
*IKZF3*	5′‐TGTTTATTAGTAAAGGAGGTTGGGATAGG‐3′	5′‐CCCTCCTAAACCATCACTTTCAAACTT‐3′
*IRF4*	5′‐CCTTTTATGCTTGTGCCCCA‐3′	5′‐GTCAGCTCCTTCACGAGGATT‐3′
*ACTB* (Acce*SssI*ble)	5′‐AGAGGGGGTAAAAAAATGTTGTAT‐3′	5′‐TCGAACCATAAAAAACAACTTTC‐3′
*C1D* (Acce*SssI*ble)	5′‐TTTTTGGAGAAGAGTTAAGGAGTAGG‐3′	5′‐ACTCCAATCTCCCGAAAAAC‐3′
*GRP78* (Acce*SssI*ble)	5′‐AGATAGTTGTTGAATTAATGGGATT‐3′	5′‐CCCCCCAACTAATTCATTAACTA‐3′

### 2.7 Immunoblotting

Whole‐cell lysates were isolated from all cell lines using lysis buffer (RIPA buffer) with the addition of a combination of protease inhibitors [ethylenediaminetetraacetic acid, a protease inhibitor cocktail tablet (Roche), and phenylmethylsulfonyl fluoride]. Protein concentration was determined by generating a 6‐point standard curve using Bradford assay in duplicate. Equal amounts of protein (50 μg) were then loaded to SDS/PAGE gels, followed by transfer to a polyvinylidene difluoride membrane. After blocking with 5% BSA in TBS‐T, membranes were incubated with the relevant primary antibody overnight at 4 °C, washed, and incubated for 1 h with a HRP‐conjugated anti‐rabbit secondary antibody (Abcam, Cambridge, UK). Proteins were detected using chemical luminescence (ECL Plus™ Chemiluminescent Detection System, Thermo Fisher Scientific, Waltham, MA, USA). The following primary antibodies were used in this study: rabbit polyclonal CRBN antibody (Sigma, St. Louis, MO, USA; cat.no: HPA‐045910), rabbit polyclonal IKZF1 antibody (Proteintech, Rosemont, IL, USA), and secondary anti‐rabbit polyclonal H3 antibody (Abcam).

### 2.8 Cytospin and immunohistochemical staining

Cells were harvested and washed with PBS to a concentration of 4–5 × 10^6^ cells·mL^−1^. The cells were attached to individual slides by centrifugation of approximately 200 μL of cell suspension at 600 r.p.m. for 2–4 min. Following a standard procedure of fixing, washing, and blocking the attached cells, the slides were stained for CRBN using the HPA045910 antibody from Sigma, at a 1 : 1000 dilution. The stained slides were finally microscopically evaluated and scored independently by two hematopathologists, with a cutoff of 30% used for CRBN positivity.

### 2.9 Acce*SssI*ble assay

The principle of this assay has already been described and published, but we slightly modified the protocol for our cell lines (Pandiyan *et al*., 2013). Briefly, 500 000 cells in exponential growth phase were harvested and split into two microcentrifuge tubes (no enzyme (NoE) and M.SssI reactions; 250 000 cells per reaction), centrifuged at approximately 200 ***g*** for 5 min, and then resuspended in 60 μL PBS. For nuclei isolation, 1 mL of lysis buffer [10 mmol·L^−1^ Tris (pH 7.4), 10 mmol·L^−1^ NaCl, 3 mmol·L^−1^ MgCl_2_, 0.1 mmol·L^−1^ EDTA, 0.5% NP‐40] was added, and the cells were centrifuged at approximately 700 ***g*** for 5 min at 4 °C after an incubation of approximately 10 min on ice. The supernatant was removed and the nuclear pellets were resuspended in 1 mL wash buffer [10 mmol·L^−1^ Tris (pH 7.4), 10 mmol·L^−1^ NaCl, 3 mmol·L^−1^ MgCl_2_, 0.1 mmol·L^−1^ EDTA] and centrifuged again at 3000 r.p.m. for 5 min at 4 °C. The supernatant was removed and the following was added to each tube: 76.75 μL 1× NEB buffer 2, 7.5 μL 10× NEB buffer 2, 45 μL 1 mol·L^−1^ sucrose, 5 μL 32 mmol·L^−1^ S‐adenosylmethionine (SAM), and 15 μL 4 U·μL^−1^ M.SssI (or H_2_O for NoE tube). The reaction mixtures were flicked to mix and then incubated at 37 °C for 7.5 min. An additional 5 μL of SAM was added and the samples were incubated for further 10 min. Prewarmed (37 °C) 300 μL Stop Solution [10 mmol·L^−1^ Tris/HCl (pH 7.9), 600 mmol·L^−1^ NaCl, 1% SDS, 0.1 mmol·L^−1^ EDTA] and 3 μL Proteinase K (20 mg·mL^−1^) were added to each tube, and each reaction mixture was incubated at 55 °C for 16 h. The DNA was then purified by phenol/chloroform extraction and ethanol precipitation and finally redissolved in 21 μL nuclease‐free water for the subsequent analyses. One microgram of DNA was bisulfite‐converted using the Zymo EZ DNA Methylation Kit, and subsequent quality control of M.SssI treatment was performed as previously described (Becket *et al*., 2016), using ACTB, C1D, and GRP78 as controls. The primer sequences are given in Table 1.

### 2.10 ChIP assay

For ChIP, we harvested 1 × 10^7^ exponentially growing cells, fixed them at room temperature using 1% formaldehyde, and then sonicated the fixed cells for 30 s with 30 s of pause on ice, for a total of nine cycles. Chromatin size was tested by running the DNA in a 1.5% agarose gel, and protein content was quantified using Bradford assay. The samples were then incubated with the desired primary antibody and beads overnight at 4 °C. After washing and de‐cross‐linking the following day, DNA was purified using PCR columns (Qiagen), diluted 1 : 2, and then qPCR was performed in duplicate, normalizing to a biological duplicate of total input.

### 2.11 Genome‐wide methylation and chromatin accessibility analysis

The Infinium HumanMethylationEPIC BeadChip array (Illumina, Inc., San Diego, CA, USA), containing a total of 866 836 probes, was used to analyze genome‐wide DNA methylation patterns. Data import and analysis, normalization, background and dye bias correction, and calculation of β values were performed using the R package minfi (Fortin *et al*., 2016). Probes containing a SNP (*N* = 340 327), cross‐reactive probes (*N* = 42 558) (both based on McCartney *et al*. (2016)), probes located on the sex chromosomes (*N *= 19 681), and probes with a high detection *P*‐value (above 0.01) were filtered before further analyses, leaving a total of 522 554 probes.

Accessibility for each cell line (Acc) was defined as the difference between the corrected β‐values (range 0–1) of the M.SssI‐treated sample and of the NoE sample (endogenous methylation values for each CpG locus). After removing negative values of Acc for each cell line, Acc and NoE values of every resistant cell line were then subtracted from their respective values in their sensitive counterpart, resulting in delta‐methylation (ΔMeth) and delta‐accessibility (ΔAcc) values. ΔMeth and ΔAcc values (Δβ values) were used in the subsequent analyses as values for changes in DNA methylation and accessibility upon acquired IMiD resistance, using a cutoff of Δβ value ± 0.20 for changes at each locus.

### 2.12 Transcriptome analysis

A total of 1 μg of RNA was used for RNA‐seq. We sequenced ten cell lines (IMiD‐sensitive OPM2 and NCI‐H929, their IMiD‐resistant counterparts, and the resensitized cells after 48 h of treatment with 5‐azacytidine and EPZ‐6438), all in technical duplicate, leaving a total of 20 samples. The sample quality control, library preparation and quality control, sequencing, and data quality control were performed by BGI. Briefly, library preparation was performed using oligo‐dT beads for enrichment with mRNA containing poly‐A tails. RNA was then fragmented and reverse‐transcribed to double‐stranded cDNA (dscDNA) using random hexamer primers. Each library was then sequenced at a depth of 20M clean reads using SE50 sequencing on the BGISEQ500 platform. The dscDNA was then end‐repaired and ligated to the bubble adapter with protruding T of 3′ end. During PCR amplification step, the fragments were separated into single strands, amplified, and cyclized to form ‘DNA nanoballs’, which were finally sequenced at a depth of 20M clean reads.

### 2.13 Transcriptome data analysis

RNA‐seq data were aligned to the human reference genome (GRCh37/hg19) using Tophat, as part of the cufflinks pipeline. Aligned reads were then assembled into transcripts and quantified using the cufflinks pipeline (Trapnell *et al*., 2012). Raw transcript counts, as produced by the cuffnorm step of cufflinks, were then processed using the R package DESeq2 in order to discover differentially expressed genes across sample groups. Differential expression was determined without the use of a Cooks cutoff, and differentially expressed genes were considered significant based on both their fold change (absolute value of log2‐fold change above 1) and their adjusted *P*‐value using the Benjamini–Hochberg method (*P* < 0.05).

### 2.14 Statistical analysis

All the statistical analyses and calculations for Acce*SssI*ble and RNA‐seq were performed in the statistical software r (version 3.3.1, https://www.r-project.org/). Comparison of mean values across different subgroups was made by ANOVA (making the assumption that the data were normally distributed for each group), with the analyses performed in graphpad prism 6. When multiple comparisons were made, the *P*‐values were corrected with the Bonferroni method. The level for significance was set at 0.05 with the following annotation: *P < 0.05, **P < 0.01, ***P < 0.001, and ****P < 0.0001.

## 3 Results

### 3.1 Cereblon is downregulated in acquired IMiD resistance

**Figure 1:**
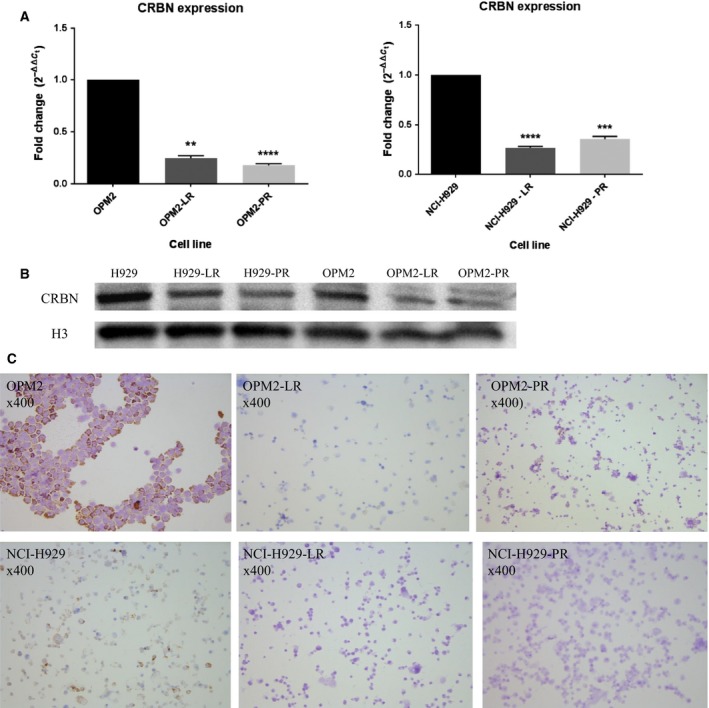
(A) qPCR results of the CRBN expression across the IMiD‐sensitive cell lines (OPM2, NCI‐H929: dark bars on the left and right plot, respectively) and their lenalidomide‐ and pomalidomide‐resistant counterparts (dark gray and light gray bars, respectively). There is a significant downregulation of CRBN mRNA in all four cell lines with acquired IMiD resistance, compared to the parental, sensitive cell lines. **P < 0.01, ***P < 0.001, and ****P < 0.0001.(B) Western blot for CRBN, confirming the reduction in CRBN expression at protein level in loss of IMiD sensitivity. (C) Cytospin and immunohistochemical staining for CRBN in OPM2, NCI‐H929, and their IMiD‐resistant counterparts, confirming the significant reduction in CRBN expression in the resistant cells.

First, we tested the sensitivity of all the cell lines to both lenalidomide and pomalidomide by generating dose–response curves and calculating the IC_50_ of both drugs for each cell line. Using the two most IMiD‐sensitive cell lines, namely OPM2 and NCI‐H929, we were able to generate lenalidomide‐ and pomalidomide‐resistant cell lines (OPM2‐LR, OPM2‐PR and NCI‐H929‐LR, NCI‐H929‐PR, respectively) by continuously culturing the cells in the presence of IMiDs for a period of 4–6 months (Fig. S1A,B). Consistent with previously published data, we observed a significant reduction in CRBN expression in all four IMiD‐resistant cell lines when compared with their sensitive counterparts at mRNA level, using qPCR primers including exons 8 and 9 (Fig. 1A) and exons 10 and 11 (the IMiD‐binding region of CRBN – data not shown), both producing similar results. For the protein expression of CRBN, we performed western blotting, as well as immunohistochemical staining for CRBN for the cell lines. We observed a decrease in the protein expression of CRBN in IMiD‐resistant cell lines using both methods, consistent with the reduction in mRNA levels (Fig. 1B,C). No significant expression changes in IKZF1, IKZF3, or IRF4 were observed (data not shown).

### 3.2 Cereblon expression is not regulated by promoter methylation

**Figure 2:**
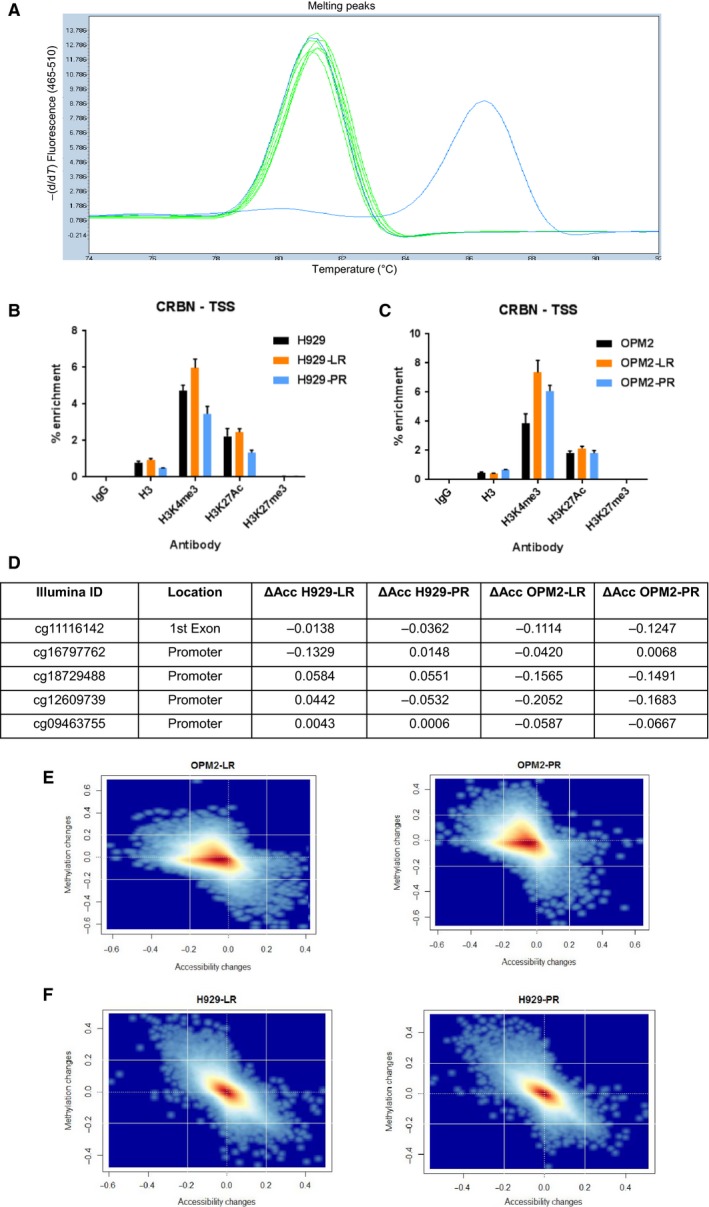
(A) Methylation‐specific melting curve analysis for the IMiD‐sensitive and IMiD‐resistant cell lines, showing the absence of promoter DNA methylation of CRBN. The blue curves are the fully unmethylated and fully methylated controls (left and right curve, respectively), and the green lines represent the cell samples. (B, C) ChIP for H3K4me3, H3K27Ac, and H3K27me3 around the transcription site of CRBN for OPM2 and NCI‐H929, respectively, revealed that IMiD‐resistant cell lines retained their activating histone marks (H3K4me3, H3K27Ac) without any significant increase in the repressive mark H3K27me3. (D) Chromatin accessibility values for the probes mapping to CRBN, assessed with Acce*SssI*ble. The difference in the accessibility of the resistant cell lines compared with their parental cell line (ΔAcc) was calculated by subtracting the accessibility value of the paternal cell line from the accessibility value of the resistant cell line for each individual probe. None of the probes covering CRBN showed significant accessibility changes in any of the IMiD‐resistant cell lines. (E, F) Kernel density plot of the combined DNA methylation changes and chromatin accessibility changes observed in the IMiD‐resistant cell lines. The OPM2 IMiD‐resistant cells exhibit primarily a decrease in the global chromatin accessibility when compared with the parental OPM2 (E), while H929 IMiD‐resistant cell lines exhibit a pattern of combined loss of chromatin accessibility with increased DNA methylation (F).

Previous studies have shown that mutations in the coding sequence of CRBN are rare. In addition, in agreement with previous studies, we observed a strong downregulation of CRBN mRNA expression in IMiD‐resistant cell lines, suggesting that the major mechanism of IMiD resistance is caused by reduced transcription of CRBN. Therefore, we hypothesized that epigenetic silencing through promoter hypermethylation might be a possible mechanism explaining the downregulation of CRBN in the IMiD‐resistant cell lines. Using MS‐MCA, we tested all the cell lines used in this study, as well as a total of 48 patients with newly diagnosed MM and 41 patients with relapsed MM. None of the cell lines, sensitive or resistant, and none of the patient samples showed hypermethylation of the promoter area of CRBN (Fig. 2A and Fig. S1). Thus, these data suggest that the proximal promoter of *CRBN* is consistently unmethylated and variations in its expression are not caused by changes in DNA methylation.

### 3.3 Cereblon promoter retains active histone marks and remains nucleosome‐depleted in IMiD‐resistant cell lines

After having excluded DNA methylation as a potential epigenetic regulatory mechanism of CRBN expression, we examined whether the promoter of CRBN exhibited either an increase in the repressive histone mark H3K27me3, or an increase in nucleosome occupancy in the IMiD‐resistant cell lines. By performing ChIP for the genomic area around the transcription start site (TSS) of CRBN, we observed that in both OPM2‐ and NCI‐H929 IMiD‐resistant cells, that area maintained enrichment of histone marks associated with active transcription (H3K4me3, H3K27Ac) and was depleted of H3K27me3 (Fig. 2B,C).

Furthermore, using Acce*SssI*ble, we compared both the genome‐wide DNA methylation and chromatin accessibility changes between OPM2 and NCI‐H929 and their IMiD‐resistant counterparts. The assay covered (after filtering irrelevant probes – see 2) a total of five probes for CRBN with four in the promoter area (200 bp upstream of the transcription start site) and one in the first exon of *CRBN* gene. Confirming our previous results, none of these probes exhibited changes in methylation levels between the sensitive and resistant cell lines (data not shown). Even more interestingly, none of the probes showed significant decreases in chromatin accessibility, with all ΔAcc values for all probes being higher than the cutoff of −0.20 (Fig. 2D). Overall, these data suggest that the proximal promoter of *CRBN* is in an active conformation and that the expression of CRBN is not influenced by epigenetic mechanisms (DNA methylation, histone modifications, nucleosome occupancy) but probably through other *cis* or *trans* regulatory mechanisms.

### 3.4 Acquired resistance to IMiDs is associated with global changes in DNA methylation and chromatin accessibility

Using Acce*SssI*ble, we were able to compare the combined chromatin accessibility and methylation changes between IMiD‐sensitive cell lines for a total of 522 554 probes across the genome. Interestingly, we found that acquired resistance for both lenalidomide and pomalidomide in OPM2 was primarily associated with a global decrease in chromatin accessibility and to a much lesser extent with DNA methylation changes (Fig. 2E). However, NCI‐H929 IMiD‐resistant cells exhibited a slightly different pattern, with a combined increase in DNA methylation and decrease in chromatin accessibility (Fig. 2F). Slightly less than half of these changes occurred in promoter areas of known genes (Fig. S2A). Interestingly, none of the other molecules involved in the CRBN pathway (IKZF1, IKZF3, IRF4) exhibited changes in either promoter DNA methylation or chromatin accessibility, which was not surprising, as their expression remained unchanged upon acquired IMiD resistance. Thus, these data suggest that resistance to IMiDs is associated with extensive epigenetic reprogramming that includes changes in chromatin accessibility and DNA methylation, but these events do not directly involve the core components of the CRBN pathway.

### 3.5 Acquired resistance to IMiDs is potentially reversible with epigenetic therapy

**Figure 3:**
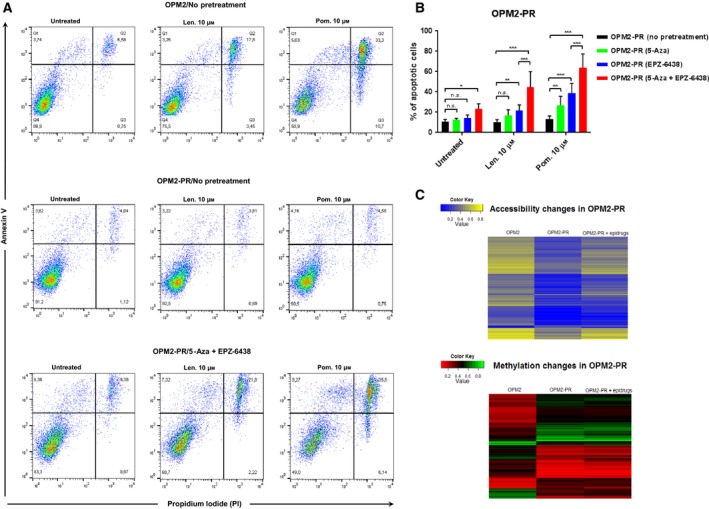
(A) Apoptosis results for OPM2, OPM2‐PR, and resensitized OPM2‐PR. The original OPM2 cells are sensitive to both lenalidomide and pomalidomide, where treatment with 10 μm of either compound led to an apoptotic response after 72 h. In contrast, both drugs fail to induce apoptosis in the pomalidomide‐resistant OPM2‐PR cells. However, after epigenetic pretreatment with 5‐Aza (0.1 μm) and EPZ‐6438 (10 μm) for 48 h and subsequent treatment with either lenalidomide or pomalidomide for 3 days, the apoptotic response to the drugs was restored. (B) Apoptosis measurements for OPM2‐PR without any pretreatment (black bars), with pretreatment only with 0.1 μm of 5‐Aza (green bars), with EPZ‐6438 (blue bars), and with both (red bars). Even though both 5‐Aza and EPZ‐6438 have a slight resensitizing effect as monotherapy, the combination is significantly more effective in restoring IMiD sensitivity to OPM2‐PR cells. *P < 0.05, **P < 0.01, and ***P < 0.001. (C) Heatmaps with all the probes exhibiting either accessibility changes (*N* = 6844, upper heatmap in blue/yellow) or DNA methylation changes (*N* = 2102, bottom heatmap in red/green) in OPM2‐PR when compared with OPM2. The majority of the probes (approximately 80%) that exhibited altered chromatin accessibility restored their initial accessibility values after 48 h of treatment with 5‐Aza and EPZ‐6438, while the acquired DNA methylation patterns in the resistant cells were more stable.

As epigenetic modifications have been shown to be druggable and potentially reversible, we next examined whether it was possible to restore normal levels of chromatin accessibility and/or DNA methylation of important regulatory genomic areas by treatment with a panel of epigenetic modulators: a DNA methyltransferase inhibitor (5‐azacytidine), an HDAC inhibitor (panobinostat), and an EZH2 inhibitor (EPZ‐6438), tested both as monotherapy and in different combinations. All IMiD‐resistant cell lines were treated with different doses and combinations of these epidrugs for 48 h and then exposed to either no further treatment (control) or 10 μm lenalidomide or pomalidomide for an additional period of 3 days, after which we evaluated the resensitization effect by measuring apoptosis. Using OPM2‐PR as a model, we found that the most effective resensitization epigenetic therapy was the combination of 5‐azacytidine (5‐Aza) and EPZ‐6438, with panobinostat and EPZ‐6438 as the second most effective (Fig. S2B). Further examining the potential of this combination, we found that exposure to the combination of 5‐Aza and EPZ‐6438 for 48 h could effectively resensitize IMiD‐resistant cells with an apoptotic response very similar to the original, IMiD‐sensitive cells (Fig. 3A). The combination of 5‐Aza and EPZ‐6438 could effectively resensitize all four cell lines with acquired IMiD resistance, albeit the dose of 5‐Aza should be increased to 0.5 μm in the case of NCI‐H929‐LR and NCI‐H929‐PR for the combination to be effective, which is in line with our previous finding that NCI‐H929 cells exhibit more DNA methylation changes upon acquiring resistance to IMiDs (Fig. S2C). Even more interestingly, we found that the treatment with 5‐Aza and EPZ‐6438 almost completely restored the majority of the global chromatin accessibility changes associated with IMiD resistance back to the initial state, even though the same was not observed for DNA methylation changes (Fig. 3C, Fig. S2D). Overall, these data suggest that acquired resistance to IMiDs is mainly associated with a global epigenetic reprogramming affecting both chromatin accessibility and DNA methylation, and can potentially be restored by simultaneously inhibiting DNA methylation and EZH2.

### 3.6 Transcriptome changes in IMiD‐resistant cell lines are mainly characterized by gene downregulation

In order to examine whether the observed changes in chromatin accessibility and DNA methylation across the IMiD‐resistant cell lines coincide with aberrant gene expression patterns, we performed RNA‐seq analysis in the sensitive, resistant, and resensitized cell lines. We found that IMiD‐resistant cell lines indeed exhibit altered global gene expression – primarily downregulation (Fig. S3A,B), consistent with the epigenetic changes we observed in these cells with a predominance of reduced chromatin accessibility and increased DNA methylation. Interestingly, the overlap of known genes with significantly increased DNA methylation and/or reduced chromatin accessibility and of genes with reduced gene expression in the resistant cell lines was rather small (Fig. 3C). When we isolated the genes whose expression was restored after treatment with 5‐Aza and EPZ‐6438, we identified *SMAD3* as a commonly downregulated gene across all four IMiD‐resistant cell lines with subsequent upregulation and normalization of expression after epigenetic resensitization (Fig. 3D). Overall, our data suggest that acquired IMiD resistance is also associated with global gene expression changes, although in a lesser degree than epigenetic changes, and identify *SMAD3* as a gene whose expression might play a significant role in the sensitivity of plasma cells to IMiDs.

### 3.7 Resensitization to IMiDs is independent of CRBN

**Figure 4:**
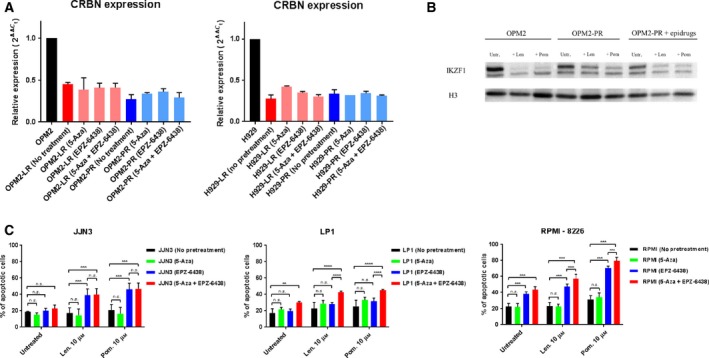
(A) The expression of CRBN at mRNA level was not restored after treatment of the resistant cells with 5‐Aza, EPZ‐6438, or their combination, suggesting that the downregulation of CRBN, which is observed in all the IMiD‐resistant cells with acquired resistance, might not have a direct causality to the development of resistance. (B) Western blot of IKZF1 in OPM2, OPM2‐PR, and OPM2‐PR cells after treatment with 5‐Aza and EPZ‐6438. All the cells were either untreated or treated with lenalidomide or pomalidomide (10 μm) for 24 h, at which point cell lysates were isolated. The CRBN‐dependent degradation of IKZF1 after treatment with IMiDs is reduced in OPM2‐PR, but is partly rescued in the resensitized OPM2‐PR cells. (C) Apoptosis response in primary IMiD‐resistant cell lines JJN3, LP1, and RPMI‐8226, showing that the combination of 5‐Aza and EPZ‐6438 – or EZH2 inhibition alone (in the case of JJN3), can also overcome intrinsic resistance to IMiDs. **P < 0.01, ***P < 0.001, and ****P < 0.0001.

As the resistant cell lines exhibited a significant decrease in CRBN expression potentially due to the observed genome‐wide increase in nucleosome occupancy, we decided to examine whether the combination of 5‐Aza and EPZ‐6438 was able to restore the levels of CRBN expression back to normal. As expected, monotherapy with each of the drugs failed to result to an increase in CRBN expression (Fig. 4A). However, more interestingly, we observed that treatment with the combination of 5‐Aza and EPZ‐6438 also failed to induce a significant upregulation of CRBN, thus suggesting that the process of resensitization to IMiDs might be CRBN independent. Furthermore, we found that degradation of IKZF1, 24 h after treatment with either lenalidomide or pomalidomide, was abrogated in OPM2‐PR, but was partly restored after the cells were treated with 5‐Aza and EPZ‐6438 (Fig. 4B). This finding not only confirms that degradation of IKZF1 might be essential for the antimyeloma effect of IMiDs, but also suggests that low expression of CRBN does not necessarily translate to reduced sensitivity to IMiDs.

### 3.8 Intrinsic resistance to IMiDs is potentially also reversible through EZH2 inhibition

Even though intrinsic resistance to IMiDs is probably biologically distinctive from acquired resistance, we tested the resensitizing efficacy of 5‐Aza and EPZ‐6438 in eight primary resistant or partly resistant IMiDs cell lines: JJN3, RPMI‐8226, U266, KMS12‐BM, MOLP2, MOLP8, LP1, and EJM. We found that 5‐Aza (doses ranging from 0.1 to 0.5 μm) and EPZ‐6438 (doses ranging from 1 to 10 μm) in combination significantly increased the sensitivity of cells to both lenalidomide and pomalidomide in seven of eight cell lines (Fig. 4C and Fig. S4). The only cell line that remained resistant after epigenetic pretreatment was U266, which is known to carry a multidrug‐resistant phenotype. However, as a reflection of the differential mechanisms that govern intrinsic IMiD resistance, a proportion of the resistant cell lines obtained a similar degree of IMiD sensitivity with EZH2 inhibition alone, possibly suggesting that primary resistance to IMiDs might be epigenetically less complex and potentially easier to overcome.

## 4 Discussion

The discovery of CRBN as the direct biological target of all IMiDs has led to a deeper understanding of their complex mechanism of action. Early data underscored the essential role of CRBN for response to IMiDs, as cell lines with abrogated expression of CRBN lost their sensitivity to the compounds (Lopez‐Girona *et al*., 2012; Zhu *et al*., 2011). Further research has suggested that CRBN might be a predictive biomarker of response to IMiDs (Broyl *et al*., 2013; Heintel *et al*., 2013; Huang *et al*., 2014; Ren *et al*., 2015). However, lack of standardized techniques for the precise measurement of CRBN expression and broad biological variability, such as multiple transcript isoforms with potentially different roles, might explain why CRBN expression is not used as a biomarker in clinical praxis yet. In the study by Gandhi *et al*. (2013b), baseline expression of CRBN was not found to be associated with responsiveness to IMiDs and a reduced expression of CRBN was only observed during acquired loss of sensitivity to IMiDs. Our data confirmed this finding, as we observed a significant decrease in both mRNA and protein levels of CRBN in cell lines with acquired resistance to lenalidomide and pomalidomide, achieved through prolonged exposure to the agents. Interestingly, baseline levels of CRBN or any of its downstream targets did not seem to be associated with IMiD sensitivity of the cell lines used in this study, thus again questioning the predictive role of CRBN to IMiD responsiveness.

The regulatory mechanisms that govern the expression of CRBN have not yet been discovered. Early studies using NGS‐based methods showed that mutations of the CRBN‐coding sequence are rare in MM (Egan *et al*., 2013; Thakurta *et al*., 2014). However, a more recent study by Kortüm *et al*. (2016), using targeted sequencing in a cohort of 50 relapsed/refractory MM patients, found a 12% (6/50) frequency of CRBN mutations, some of which affected the IMiD‐binding domain in exons 10–11. To uncover a possible epigenetic component in the regulation of CRBN, we assessed DNA methylation, histone modifications, and nucleosome positioning of the proximal promoter area of CRBN, and showed that CRBN is not directly regulated by any of them, but likely by other *cis* or *trans* regulatory elements.

Using Acce*SssI*ble, a method that allows measurement of the global levels of both DNA methylation and chromatin accessibility, we showed that acquired IMiD resistance was accompanied by global epigenetic changes in form of decreased chromatin accessibility and increased DNA methylation. It must be mentioned here that Acce*SssI*ble is limited by the probe coverage that Illumina EPIC assay offers (approximately 500 000 probes after filtering), which means that it does not offer information about the entire genome. However, it is still a high‐throughput, but at the same time cost‐effective and bioinformatically manageable method, covering the most important regulatory genomic areas, such as gene promoters and enhancers.

Chromatin accessibility has consistently been shown to affect gene expression (Portela and Esteller, 2010). In a more recent study, it was shown to be even more cell type specific than gene expression and changes in accessibility were most prominent in disease‐specific cells in both benign and malignant diseases (Corces *et al*., 2016). In our study, we find that chromatin accessibility might play an important role in drug resistance, a finding that requires further investigation, but which is of extreme interest, as epigenetic modifications are potentially reversible.

Drugs that modify the epigenome, such as 5‐azacytidine or panobinostat, have already been approved for the treatment of some hematological malignancies (high‐risk myelodysplastic syndromes and MM, respectively). Despite the impressive *in vitro* efficacy of both 5‐azacytidine and HDAC inhibitors in MM, shown in several studies (Kiziltepe *et al*., 2007; Maiso *et al*., 2006; Ocio *et al*., 2010; Sanchez *et al*., 2011; Tian *et al*., 2013), their performance in clinical studies, when used as monotherapy, has been quite disappointing. Importantly, both classes of drugs are not as cytotoxic as traditional chemotherapeutic agents, but when used in low doses alone or in combination, might ‘prime’ the epigenome and enhance sensitivity to other, more toxic compounds.

In another interesting finding, we were able to inverse chromatin accessibility with a combination of 5‐azacytidine and EPZ‐6438 and restore sensitivity to both lenalidomide and pomalidomide in all four cell lines with acquired IMiD resistance. This combination was shown to almost entirely reverse the chromatin accessibility to the initial state and restore sensitivity, and interestingly, it was shown to be effective in inducing apoptosis independently of CRBN expression. This is, to our knowledge, the first report on combined DNA methyl transferase 1 (DNMT1) and EZH2 inhibition and its effects on epigenetic reprogramming and drug resistance.

The justification for this combination is extensively supported by previous data. In a study from our group, we showed that decreased chromatin accessibility in malignant cells (with a concomitant decrease in gene expression) was only partly associated with increased DNA methylation on the same probes, suggesting that the probes without DNA methylation most possibly obtain the repressive H3K27me3 histone mark (Becket *et al*., 2016). Another study showed that malignant cells can have almost double as many combined DNA methylation and H3K27me3 silencing marks, as compared to normal cells (Takeshima *et al*., 2014), further supporting treatment strategies that combine inhibition of DNMT1 and EZH2. Finally, patients with myelodysplastic syndromes (MDS) presenting with monosomy 7 (where EZH2 is located) or inactivating point mutations of EZH2 respond better to treatment with 5‐azacytidine, suggesting that dual inhibition of EZH2 and DNA methylation might be more effective in these patients (Tobiasson *et al*., 2016).

Global gene expression analysis of IMiD‐resistant cell lines showed a dominance of gene downregulation, consistent with the results from Acce*SssI*ble that showed a tendency toward increased DNA methylation and decreased chromatin accessibility, both of which are known to repress gene expression. However, the overlap between the epigenetically affected genes, discovered by Acce*SssI*ble, and the deregulated genes by RNA‐seq was rather little. The lack of direct correlation of epigenetic aberrations and gene expression could be due to the existence of noncoding genomic regions in the Illumina array, some of which might have important regulatory abilities. Furthermore, the lack of inhibitory epigenetic marks such as DNA methylation or nucleosome occupancy is permissive for, but does not alone cause gene expression, which also requires, for example, the relevant transcription factors. Finally, the Illumina probes that cover known genes do not only cover proximal regulatory areas such as promoters, but might also cover areas such as first exons or 3′‐UTR, which possibly not directly affect the expression of their corresponding gene.

The finding that *SMAD3* is commonly downregulated and subsequently upregulated by the combination therapy in all the cell lines with acquired IMiD resistance is interesting. Even though SMAD3 has not so far been directly associated with the CRBN pathway, it is a well‐known transcriptional regulator and a core component of the canonical transforming growth factor beta (TGF‐β) signaling pathway (Millet and Zhang, 2007). It has been shown that TGF‐β has an ambiguous role in tumorigenesis, enabling both growth inhibition, especially in early stages of carcinogenesis, and tumor growth in latter stages (Massagué, 2008). Accordingly, SMAD3 has been found to have both oncogenic and tumor suppressor roles in cancer. It has been shown that high expression of SMAD3 is essential for the tumor suppressive effects of TGF‐β, while lower expression levels are associated with the tumor‐promoting effect of TGF‐β (Daly *et al*., 2010). Even more interestingly, the same study showed that the levels of SMAD3 were controlled by the Ras pathway, members of which are frequently mutated in MM (Chapman *et al*., 2011). Further supporting the tumor suppressor role of SMAD3, its downregulation has been associated with acute T‐cell lymphoblastic leukemia, as well as gastric cancer (Han *et al*., 2004; Wolfraim *et al*., 2004). In addition, activation and recruitment of SMAD3 to the nucleus upon TGF‐β induction has been found to induce the cell cycle regulator p21^WAF1^ and downregulate c‐MYC (Alexandrow *et al*., 1995; Frederick *et al*., 2004), which also happen to be the end‐targets of IMiDs (Escoubet‐Lozach *et al*., 2009; Lopez‐Girona *et al*., 2011; Verhelle *et al*., 2007). A possible model might be that IMiD‐resistant plasma cells escape the antitumor effects of TGF‐β and gain a proliferation advantage by downregulating SMAD3. Otherwise, it might also be likely that the SMAD3‐dependent TGF‐β signaling is required for the IMiD‐induced downregulation of MYC and induction of p21^WAF1^ and its abrogation through downregulation of SMAD3 might reduce the effectiveness of IMiDs. However, the precise biological role of SMAD3 in IMiD resistance in myeloma and its potential use as a biomarker of responsiveness to IMiDs require further investigation.

The fact that the epigenetically induced IMiD resensitization is CRBN independent is intriguing; this might suggest that there are other direct targets for IMiDs, or it might question the role of CRBN downregulation in acquired IMiD resistance. Based on our current data, it is unlikely that CRBN downregulation is a ‘driver’ event for resistance, but rather a secondary effect of the global genomic reprogramming during the drug resistance process. Furthermore, this also means that even if acquired IMiD resistance is in some cases caused by mutations of CRBN (e.g., in the IMiD‐binding domain, as mentioned above), bypassing CRBN through epigenetic reprogramming might overcome the effect of these mutations. However, the precise mechanism through which this combination can effectively restore IMiD sensitivity has to be further inquired, as a better understanding of the complexity of the CRBN pathway and IMiD interactions may unravel novel biological targets for therapy.

In conclusion, our study is the first to ever show that acquired IMiD resistance is mainly an epigenetic event that is potentially reversible through a combination of two epigenetic compounds, 5‐azacytidine and EPZ‐6438. These drugs have been shown to have relatively low levels of toxicity, thus making them very good candidates for a prospective phase I study to examine their potential as ‘IMiD resensitizers’, which may improve the outcome of treatment of MM patients with drug‐resistant myeloma clones and a potentially high‐risk disease.

## Author contributions

KD designed this study, collected and analyzed the data, and wrote the manuscript. ASH, JC, LSK, FA, and NEUH contributed with data collection for this study. HFMP and LS contributed with data analysis for this study. COC, PG, GL, and KG were senior supervisors of this study and contributed to the design of this study, data interpretation, and preparation of the manuscript.

## Supporting information


**Fig. S1.** (A, B) Dose‐response curves for lenalidomide and pomalidomide in (A) OPM2 and (B) NCI‐H929, as well as their resistant counterparts. (C, D) Methylation specific melting curves for the CRBN promoter of CD138+ plasma cells from (C) 48 patients with newly diagnosed MM and (D) 41 patients with relapsed MM. The blue curves represent the unmethylated (left), methylated (right) and 50% methylated (bimodal) controls, while the green curves, which all fall under the unmethylated control, represent the DNA samples from the patients. The promoter of CRBN was unmethylated in all the patients analyzed in this study.
**Fig. S2.** (A) Proportional genomic distribution (promoters vs non.promoter areas) of all the probes with accessibility or DNA methylation changes across all IMiD‐resistant cell lines. A bit less than half of the probes showing significant changes in either accessibility or DNA methylation in the resistant cell lines map to promoter areas of known genes. (B) Apoptosis response of OPM2‐PR to either no treatment or 10 μm of lenalidomide or pomalidomide for 72 h, followed by a 48 h pretreatment with different epigenetic drugs. The most effective combination in restoring the apoptotic effect of IMiDs to the resistant OPM2‐PR cells was 5‐Azacytidine and EPZ‐6438. (C) Apoptotic response of H929‐PR without any pretreatment (black bars), with pretreatment only with 0.5 μm of 5‐Aza (green bars), with EPZ‐6438 (blue bars) and with both (red bars). The combination of 5‐Aza and EPZ‐6438 is effective in resensitizing the H929‐IMiD‐resistant cells in a similar manner to OPM2‐LR and OPM2‐PR. (D) Kernel density scatter plot of the accessibility changes (*x* axis) and DNA methylation changes (*y* axis) in OPM2‐PR treated with 5‐Aza and EPZ‐6438 for 48 h, compared to the paternal OPM2. The cluster of probes exhibiting decreased accessibility observed in OPM2‐PR (Fig. 2E) is significantly decreased, with more probes showing increased accessibility and decreased methylation.
**Fig. S3.** (A, B) Volcano plots of differentially expressed genes for OPM2‐PR (A) and H929‐PR (B) compared to their paternal cell lines. The dots in red represent the differentially expressed genes with an absolute value of log2 fold change above 1 and an adjusted *P*‐value (Benjamini–Hochberg method) below 0.05. There is a slight predominance of downregulated genes in both cases, supporting the data from AcceSssIble. (C) Venn diagram of all the Illumina probes mapping to known genes showing either decreased accessibility or increased DNA methylation in OPM2‐PR and all the downregulated genes found in RNA‐seq of OPM2‐PR, showing only little/some overlap between epigenetic deregulation and gene expression. (D) Expression pattern of SMAD3 in OPM2 (IMiD sensitive), OPM2‐LR and OPM2‐PR (IMiD resistant) and the epigenetically resensitized OPM2‐LR and OPM2‐PR, shown in normalized counts for gene length (fpkm: Fragments Per Kilobase of transcript per Million mapped reads). SMAD3 follows the same expression pattern in the H929 sensitive, resistant and resensitized cell lines.
**Fig. S4.** Apoptosis measurements in five primary IMiD‐resistant or partly resistant cell lines: KMS12‐BM, MOLP2, EJM, MOLP8 and U266.
