# Supplementary material for: Dual inhibition of DNMTs and EZH2 can overcome both intrinsic and acquired resistance of myeloma cells to IMiDs in a cereblon‐independent manner
Source: Mol Oncol. 2017 Dec 30;12(2):180–95. doi: 10.1002/1878-0261.12157 (PMC5792743; doi:10.1002/1878-0261.12157)
Supplement: Supplementary file 1 — Fig. S1. (A, B) Dose‐response curves for lenalidomide and pomalidomide in (A) OPM2 and (B) NCI‐H929, as well as their resistant counterparts. (C, D) Methylation specific melting curves for the CRBN promoter of CD138+ plasma cells from (C) 48 patients with newly diagnosed MM and (D) 41 patients with relapsed MM. The blue curves represent the unmethylated (left), methylated (right) and 50% methylated (bimodal) controls, while the green curves, which all fall under the unmethylated control, represent the DNA samples from the patients. The promoter of CRBN was unmethylated in all the patients analyzed in this study. Fig. S2. (A) Proportional genomic distribution (promoters vs non.promoter areas) of all the probes with accessibility or DNA methylation changes across all IMiD‐resistant cell lines. A bit less than half of the probes showing significant changes in either accessibility or DNA methylation in the resistant cell lines map to promoter areas of known genes. (B) Apoptosis response of OPM2‐PR to either no treatment or 10 μm of lenalidomide or pomalidomide for 72 h, followed by a 48 h pretreatment with different epigenetic drugs. The most effective combination in restoring the apoptotic effect of IMiDs to the resistant OPM2‐PR cells was 5‐Azacytidine and EPZ‐6438. (C) Apoptotic response of H929‐PR without any pretreatment (black bars), with pretreatment only with 0.5 μm of 5‐Aza (green bars), with EPZ‐6438 (blue bars) and with both (red bars). The combination of 5‐Aza and EPZ‐6438 is effective in resensitizing the H929‐IMiD‐resistant cells in a similar manner to OPM2‐LR and OPM2‐PR. (D) Kernel density scatter plot of the accessibility changes (x axis) and DNA methylation changes (y axis) in OPM2‐PR treated with 5‐Aza and EPZ‐6438 for 48 h, compared to the paternal OPM2. The cluster of probes exhibiting decreased accessibility observed in OPM2‐PR (Fig. 2E) is significantly decreased, with more probes showing increased accessibility and decreased methylation. Fig. S3. [file MOL2-12-180-s001.docx]

Supplementary Figures:


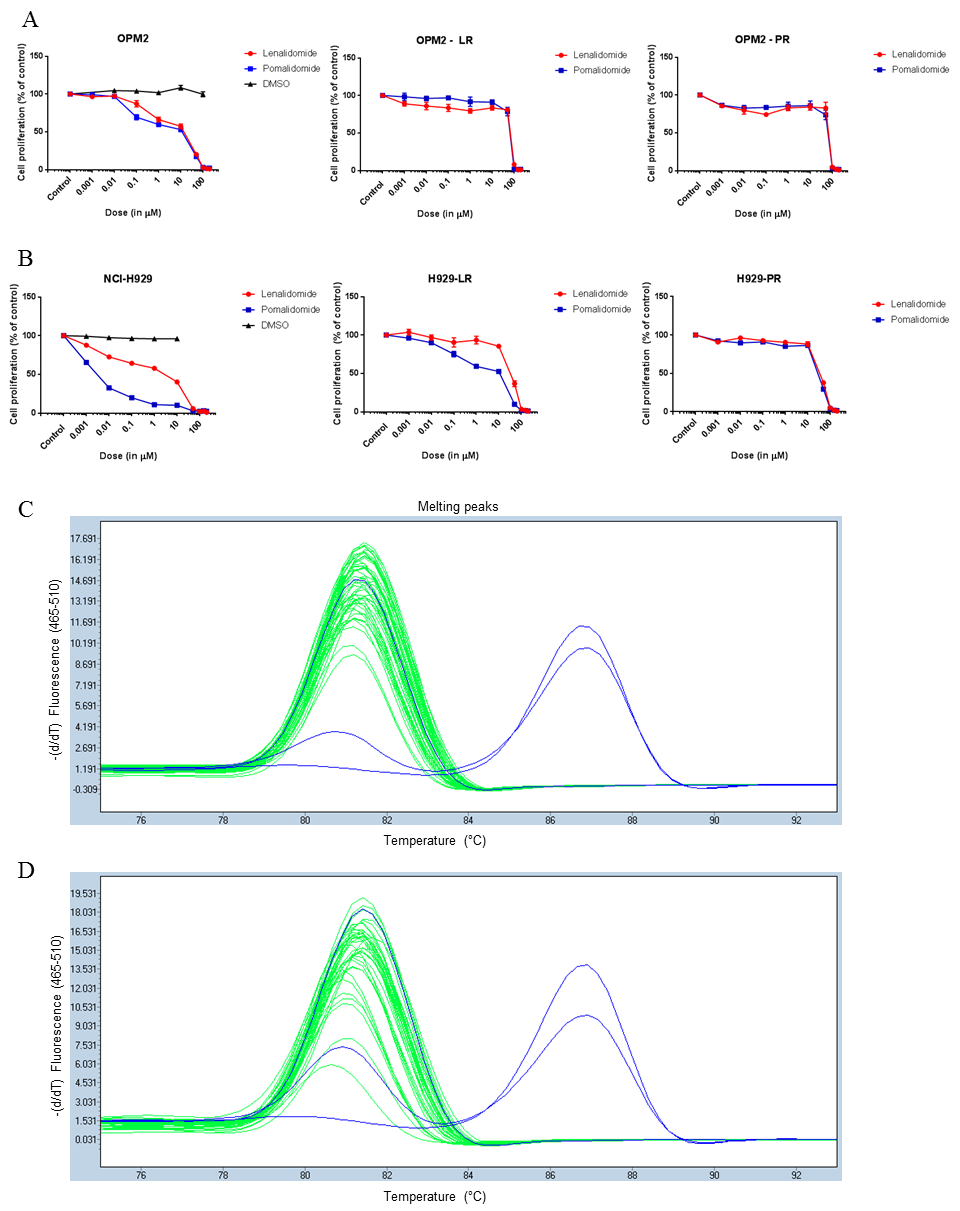


Supplementary figure 1. A, B) Dose-response curves for lenalidomide and pomalidomide in (A) OPM2 and (B) NCI-H929, as well as their resistant counterparts. C,D) Methylation specific melting curves for the *CRBN* promoter of CD138+ plasma cells from (C) 48 patients with newly diagnosed MM and (D) 41 patients with relapsed MM. The blue curves represent the unmethylated (left), methylated (right) and 50% methylated (bimodal) controls, while the green curves, which all fall under the unmethylated control, represent the DNA samples from the patients. The promoter of *CRBN* was unmethylated in all the patients analyzed in this study.


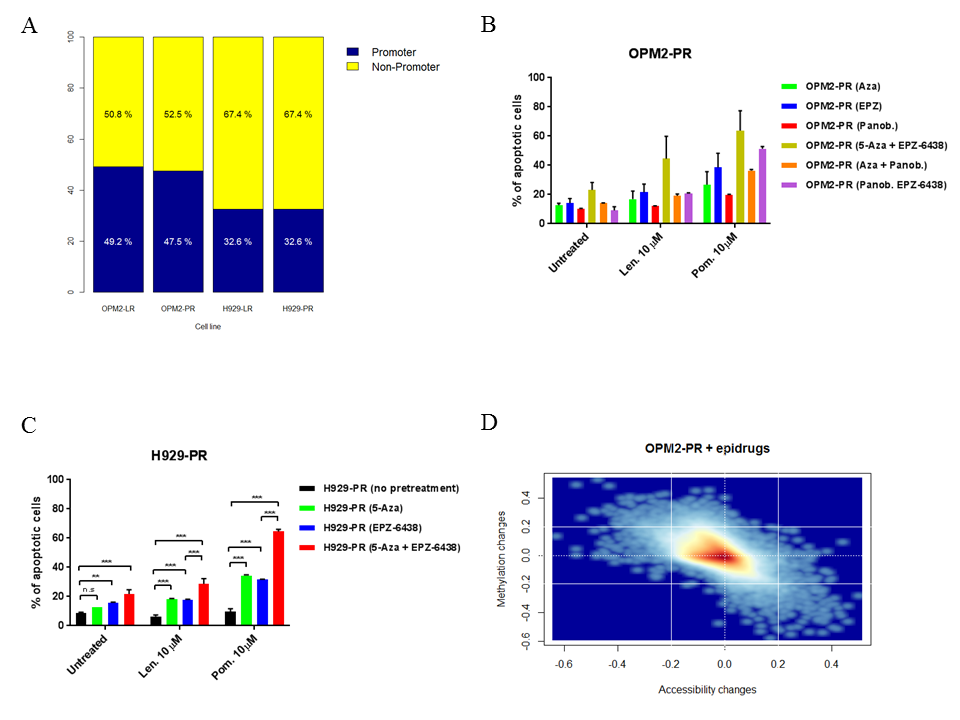


Supplementary figure 2. A) Proportional genomic distribution (promoters vs non.promoter areas) of all the probes with accessibility or DNA methylation changes across all IMiD-resistant cell lines. A bit less than half of the probes showing significant changes in either accessibility or DNA methylation in the resistant cell lines map to promoter areas of known genes. B) Apoptosis response of OPM2-PR to either no treatment or 10 μΜ of lenalidomide or pomalidomide for 72 hours, followed by a 48 hour pretreatment with different epigenetic drugs. The most effective combination in restoring the apoptotic effect of IMiDs to the resistant OPM2-PR cells was 5-Azacytidine and EPZ-6438. C) Apoptotic response of H929-PR without any pretreatment (black bars), with pretreatment only with 0.5 μM of 5-Aza (green bars), with EPZ-6438 (blue bars) and with both (red bars). The combination of 5-Aza and EPZ-6438 is effective in resensitizing the H929-IMiD-resistant cells in a similar manner to OPM2-LR and OPM2-PR. D) Kernel density scatter plot of the accessibility changes (x axis) and DNA methylation changes (y axis) in OPM2-PR treated with 5-Aza and EPZ-6438 for 48 hours, compared to the paternal OPM2. The cluster of probes exhibiting decreased accessibility observed in OPM2-PR (figure 2E) is significantly decreased, with more probes showing increased accessibility and decreased methylation.


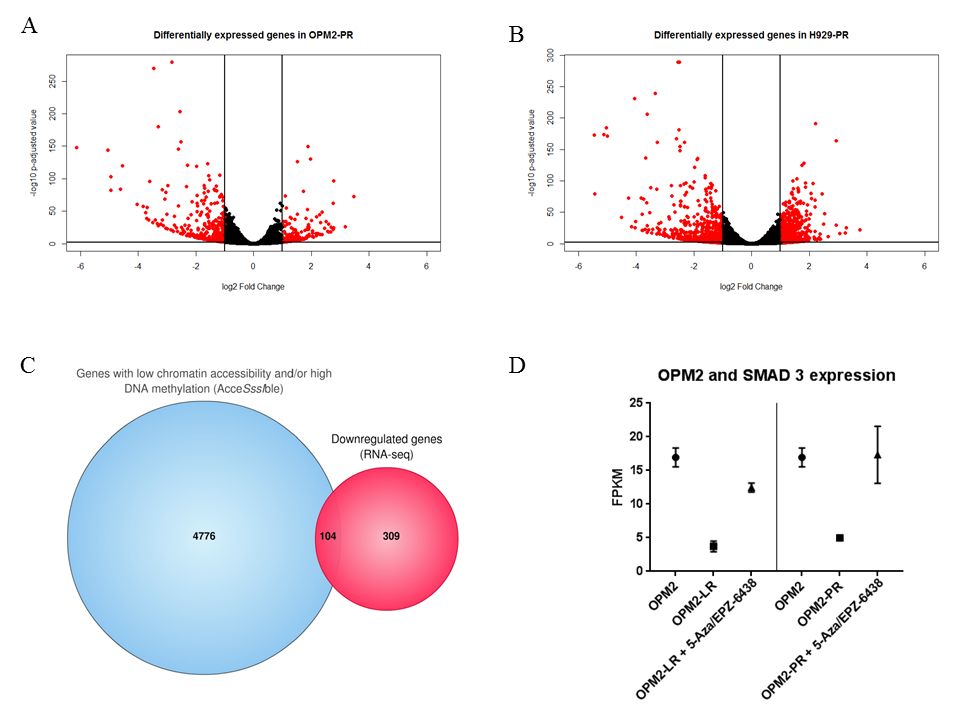


Supplementary figure 3. A,B) Volcano plots of differentially expressed genes for OPM2-PR (A) and H929-PR (B) compared to their paternal cell lines. The dots in red represent the differentially expressed genes with an absolute value of log2 fold change above 1 and an adjusted p-value (Benjamini-Hochberg method) below 0.05. There is a slight predominance of downregulated genes in both cases, supporting the data from Acce*SssI*ble. C) Venn diagram of all the Illumina probes mapping to known genes showing either decreased accessibility or increased DNA methylation in OPM2-PR and all the downregulated genes found in RNA-seq of OPM2-PR, showing only little/some overlap between epigenetic deregulation and gene expression. D) Expression pattern of SMAD3 in OPM2 (IMiD sensitive), OPM2-LR and OPM2-PR (IMiD resistant) and the epigenetically resensitized OPM2-LR and OPM2-PR, shown in normalized counts for gene length (fpkm: Fragments Per Kilobase of transcript per Million mapped reads). SMAD3 follows the same expression pattern in the H929 sensitive, resistant and resensitized cell lines.


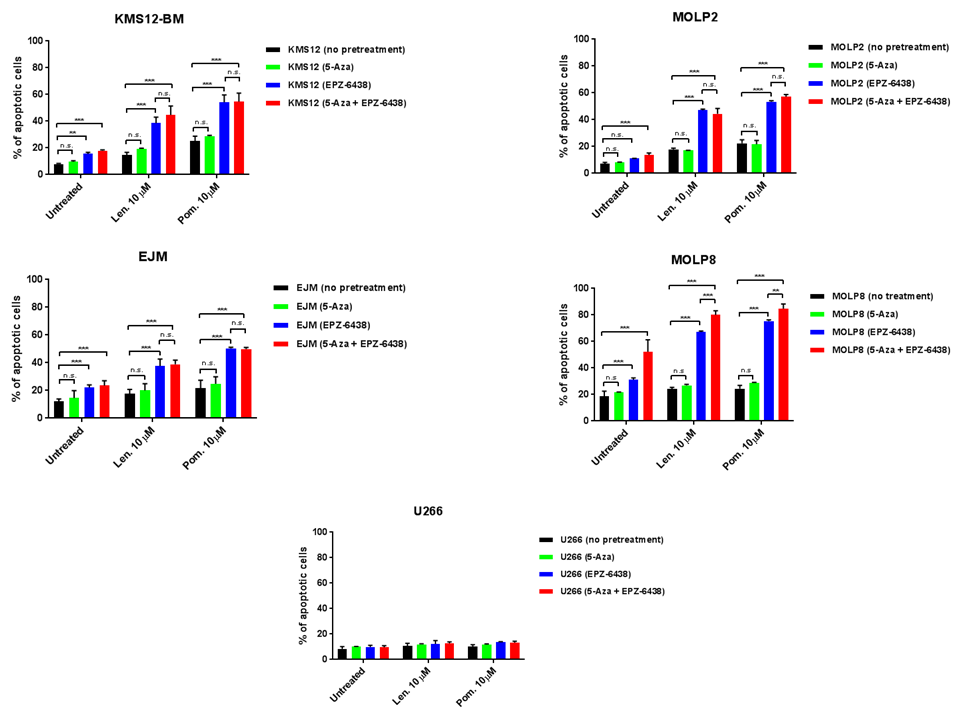


Supplementary figure 4. Apoptosis measurements in five primary IMiD-resistant or partly resistant cell lines: KMS12-BM, MOLP2, EJM, MOLP8 and U266. A total of eight cell lines with intrinsic resistance to lenalidomide and pomalidomide were treated with 5-Aza and EPZ-6438, with seven of them becoming sensitive to both drugs. The epigenetic treatment was itself slightly toxic for some of the cell lines (eg. MOLP8, EJM), while in some cases, EPZ-6438 as monotherapy was sufficient in sensitizing the cells to IMiDs.
